# Human Mesenchymal Stem Cells Reendothelialize Porcine Heart Valve Scaffolds: Novel Perspectives in Heart Valve Tissue Engineering

**DOI:** 10.1089/biores.2015.0019

**Published:** 2015-06-01

**Authors:** Paola Lanuti, Francesco Serafini, Laura Pierdomenico, Pasquale Simeone, Giuseppina Bologna, Eva Ercolino, Sara Di Silvestre, Simone Guarnieri, Carlo Canosa, Gianna Gabriella Impicciatore, Stella Chiarini, Francesco Magnacca, Maria Addolorata Mariggiò, Assunta Pandolfi, Marco Marchisio, Gabriele Di Giammarco, Sebastiano Miscia

**Affiliations:** ^1^ Center for Aging Science (Ce.S.I.), “Università G. d'Annunzio” Foundation, Chieti, Italy.; ^2^ Department of Medicine and Aging Science, School of Medicine and Health Science, University “G. d'Annunzio” Chieti–Pescara, Chieti, Italy.; ^3^ StemTeCh Group, Chieti, Italy.; ^4^ Department of Experimental and Clinical Sciences, University “G. d'Annunzio” Chieti–Pescara, Chieti, Italy.; ^5^ Department of Neuroscience and Imaging, University “G. d'Annunzio” Chieti–Pescara, Chieti, Italy.

**Keywords:** endothelium, heart valve diseases, heart valve tissue engineering, WJ-MSCs

## Abstract

Heart valve diseases are usually treated by surgical intervention addressed for the replacement of the damaged valve with a biosynthetic or mechanical prosthesis. Although this approach guarantees a good quality of life for patients, it is not free from drawbacks (structural deterioration, nonstructural dysfunction, and reintervention). To overcome these limitations, the heart valve tissue engineering (HVTE) is developing new strategies to synthesize novel types of valve substitutes, by identifying efficient sources of both ideal scaffolds and cells. In particular, a natural matrix, able to interact with cellular components, appears to be a suitable solution. On the other hand, the well-known Wharton's jelly mesenchymal stem cells (WJ-MSCs) plasticity, regenerative abilities, and their immunomodulatory capacities make them highly promising for HVTE applications. In the present study, we investigated the possibility to use porcine valve matrix to regenerate *in vitro* the valve endothelium by WJ-MSCs differentiated along the endothelial lineage, paralleled with human umbilical vein endothelial cells (HUVECs), used as positive control. Here, we were able to successfully decellularize porcine heart valves, which were then recellularized with both differentiated-WJ-MSCs and HUVECs. Data demonstrated that both cell types were able to reconstitute a cellular monolayer. Cells were able to positively interact with the natural matrix and demonstrated the surface expression of typical endothelial markers. Altogether, these data suggest that the interaction between a biological scaffold and WJ-MSCs allows the regeneration of a morphologically well-structured endothelium, opening new perspectives in the field of HVTE.

## Introduction

In the past two decades, cardiac valve diseases have undergone deep changes, concerning the epidemiological, diagnostic, and therapeutic point of views. About the epidemiological profile, heart valve diseases associated to degenerative processes have become the most frequent pathologies.^1^ Regarding the diagnosis, remarkable progresses in imaging techniques allowed a more focused selection of candidates and an optimal timing for surgical correction. In such a context, echocardiography is considered the gold standard for diagnosis of heart valve diseases.^2,3^ The best therapeutic approach for their treatment is based on surgical valve replacement by mechanical or biological prosthesis implantation.^1^ Although the use of prosthesis is able to guarantee an adequate performance, this approach is characterized by short-term durability, because of structural deterioration, dysfunction, and degeneration processes of prosthesis, also complicated by hemorrhagic events due to a lifelong anticoagulant therapy.^4^ All these disadvantages often cause the need of reoperation.^5,6^ Based on such considerations, the heart valve tissue engineering (HVTE) applies specific principles to the development of substitutes able to restore, maintain, and improve sick or damaged cardiovascular structures.^6–9^ Current literature evidences the need to identify efficient sources of both ideal scaffolds and cells. Synthetic scaffolds have many advantages in terms of biocompatibility and availability, but they are made of a non-natural matrix, are expensive, potentially immunogenic, and may induce inflammation and toxic degeneration.^10–12^ Therefore, the use of a biological matrix, able to stimulate a prolonged and positive interaction with adherent cells, appears to be the most suitable solution. Concerning cell sources, many cell types are available for tissue engineering applications,^6^ and adult stem/progenitor cells seem to be the best-suited candidates in such a context; they are characterized by low risk for tumorigenesis, no evidences of side effects, and the absence of ethical issues.^13^ Among adult stem/progenitor cells, mesenchymal stem cells (MSCs), isolated from different adult tissues, show strong regenerative potential and negative immunomodulatory abilities.^14^ In particular, Wharton's jelly, the mucoid connective tissue composing the umbilical cord (UC), is emerging as an interesting source of MSCs (WJ-MSCs), since WJ-MSCs are easily available, stable for several passages, and able to differentiate along the endothelial lineage.^15,16^ Moving from these evidences, the present study evaluated the ability of WJ-MSCs and human umbilical vein endothelial cells (HUVECs)^4^ to reconstitute an efficient valve endothelium by using a decellularized porcine scaffold as a substratum for the neoendothelium growth.

## Materials and Methods

### Porcine cardiac valves

The present study was approved by the local ethical committee. Six porcine hearts were obtained from “Di Tomo Oliviero” slaughterhouse (Chieti, Italy), under veterinary supervision; the material was free from pre-existent cardiac damages. Twelve semilunar porcine cardiac valves (36 heart valve cusps) were obtained under sterile conditions. To evaluate the best protocol for decellularization and reendothelialization processes, either entire porcine heart valves or single porcine heart valve cusps were used.

### Decellularization procedure

Before performing the decellularization step, a cleaning solution comprising Dulbecco's phosphate-buffered saline (DPBS; Sigma-Aldrich, Saint Louis, MO) and antibiotics (1% penicillin/streptomycin [P/S]; Invitrogen, Carlsbad, CA) was used. To decellularize porcine cusps, two different detergent solutions, already used in the literature, were compared: 1% sodium dodecyl sulfate (SDS; Sigma-Aldrich)+0.05% NaN_3_ (Sigma-Aldrich) in DPBS 1× and 1% Triton X-100 (Sigma-Aldrich) in DPBS 1×.^17–19^ The detergent solution was replaced every 9 h during the decellularization procedure (48 h), while keeping the material in a continuous flutter. A postdecellularization cleaning procedure (DPBS+1% P/S) was performed for the following 48 h (change every 9 h).

### WJ-MSCs isolation and culture

Human UCs were used to isolate MSCs from cordon matrix. After Wharton's jelly harvesting, the tissue was crumbled, washed, and centrifuged at 250 *g* for 5 min. The pellet was suspended in Dulbecco's modified Eagle's medium (DMEM; Gibco, Paisley, Scotland) added with 2 mg/dL of collagenase IV (Sigma-Aldrich) and placed in a continuous flutter at 37°C for 16 h. After the enzymatic digestion, the specimen was washed and added with a solution containing 2.5% trypsin–EDTA (Gibco) for 30 min at 37°C. Cell suspension was obtained and the medium was changed every 3–4 days before reaching the confluence; when in subconfluence, cells were detached using trypsin–EDTA 0.05% for 5 min at 37°C, counted, and recultured at a density of 3000 cells/cm^2^.^15^

### WJ-MSCs endothelial differentiation

WJ-MSCs were incubated for 15 days in EGM-2 BulletKit (Lonza, Walkersville, MD) added by 18% of fetal bovine serum (FBS; Gibco). Endothelial phenotype was confirmed through flow cytometry and confocal microscopy.

### HUVECs isolation and culture

Human UC was cleaned in sterile conditions, cannulated at both ends with sterile needles, and clamped. Needles were connected to two taps to wash the UC from red blood cells and blood clots with DPBS+1% P/S+1% fungizone (Sigma-Aldrich). Afterward, incubation with 1 mg/mL collagenase (Sigma-Aldrich) for 3 min at 37°C was used to allow separation of endothelial cells. The umbilical vein was fulfilled with HUVECs culture medium; the specimen was then collected in a sterile tube, centrifuged for 10 min at 300 *g*, and resuspended in a flask previously treated with 1.5% gelatin (Sigma-Aldrich). After reaching confluence, cells were detached and cultured in Medium 199 (M199; PAA, Cölbe, Germany) added with 50 μg/mL endothelial cell growth factor (ECGF; Sigma-Aldrich) and 10 μg/mL heparin (Sigma-Aldrich).^20,21^

### WJ-MSCs and HUVECs immunophenotyping

WJ-MSCs and HUVECs, respectively, at the eighth and the sixth passage, were treated with 0.05% trypsin–EDTA and collected; 10^6^ cells per sample were incubated with 1 μg of the specific antibody, conjugated with fluorescein isothiocyanate (FITC), phycoerythrin (PE), allophycocyanin (APC), phycoerythrin-cyanine 5.5 (PE Cy5.5), or Alexa Fluor 488 for 30 min at 4°C in the dark. WJ-MSCs were stained using the following antibodies: anti-CD31, anti-CD73, anti-CD13, anti-CD90, anti-CD117, anti-CD14, anti-CD34, anti-CD105, anti-CD146, anti-CD133, anti-CD144, anti-ESA, anti-HLA-ABC, anti-HLA-DR, anti-CD45 (Becton Dickinson [BD], San Jose, CA), anti-CD29, anti-CD44, and anti-CD166 (Ancell, Bayport, MN). HUVECs were stained with anti-CD146 (BD) and anti-CD144 (Acris Antibodies, San Diego, CA). After incubation, cells were washed and acquired with a flow cytometer (FACS Calibur; BD), collecting 10,000 events per sample. Data were analyzed by the FlowJo software v8.8.6 (TreeStar, Ashland, OR). The mean fluorescence intensity (MFI) ratio values were calculated (i.e., dividing the MFI of positive events by the MFI of negative events).^22,23^

### Reendothelialization

Decellularized cusps and heart valves were placed in incubator for 24 h at 37°C with 95% O_2_/5% CO, using two types of culture media: HUVECs culture medium, composed of 50% DMEM low glucose (PAA) and 50% M199 (PAA), added with 1% P/S, 1% l-glutamine (l-Glu; PAA), 20% FBS, 1% heparin (Sigma-Aldrich), and 1% ECGF (Sigma-Aldrich); EGM-2 BulletKit culture medium added by 18% FBS.

Cusps were recellularized for 15 days by HUVECs (sixth passage) or WJ-MSCs previously differentiated for 15 days in EGM-2 (eighth passage); in both cases, cells were seeded at the concentration of 1×10^6^ cells/mL. The culture medium was changed every 48–72 h.

### Heart valve cusps immunocytochemical analysis

#### Confocal microscopy analyses

Cusps (before or after the decellularization/reendothelialization processes) were fixed by 4% paraformaldehyde (Merck, Darmstadt, Germany) for 45 min and nuclei were stained by 0.05 μL/mL 4′,6-diamidino-2-phenylindole (DAPI; Sigma-Aldrich) or by DRAQ5 (1:250; Biostatus Limited, Leicestershire, United Kingdom). Cups were stained by anti-CD146-PECy7 (1:20), anti-CD144 or by anti-CD31 (1:40 dilution; BD) CD144 and CD31 were stained by an anti-mouse Alexa Fluor 546 (1:100 dilution; Life Technologies, Carlsbad, CA). Images were acquired using a confocal microscope (LSM510 META; Carl Zeiss, Oberkochen, Germany), equipped with a reverse microscope (Axiovert 200) and a Plan Neofluar 40× NA 1.3 OIL lens. The excitation was obtained with an Argon laser line (488 nm) and a HeNe laser line (543 and 633 nm). The emission was collected through a primary dichroic HydroTech filter (HTF) of 488/543/633 nm and separated with META array, selecting a range from 680 to 809 nm, while FITC emission was recorded selecting a band pass (BP) filter of 505–550 nm. DRAQ5 emission was recorded through a conventional photomultiplier using a longpass filter (650 nm) and Alexa Fluor 546 through a band pass (BP) filter of 560–615 nm. To prevent overlaps in fluorescence emissions, images were acquired sequentially.^24^ For the acquisition of DAPI-stained cusp pictures, a fluorescence microscope (50iEclipse; Nikon, Shinjuku, Japan) was used, and images were acquired with a Cool-SNAP*cf* digital charge-coupled device camera (PhotoMetrics, Huntington Beach, CA). Digital acquisition, processing, and analysis of fluorescence were performed by Meta Image Series 7.5 (MetaMorph, Metafluor, MetaVue) software obtained from Molecular Devices (LLC, Sunnyvale, CA).^25,26^

#### Multiphoton microscope analyses

Cusps (before or after the decellularization/reendothelialization processes) were fixed by 4% paraformaldehyde for 45 min and stained by 0.05 μL/mL DAPI and the Cell Membrane Labeling Kit PKH26 (1:250 dilution; Sigma-Aldrich). Images were acquired using a Zeiss LSM 7 MP multiphoton microscope (Carl Zeiss, Jena, Germany), equipped with upright microscope Axio-Examiner.Z1 and an objective W-Plan-Apo 20×/1.0 NA VIS-IR DIC. The two photon excitation was obtained using a Ti:Sapphire Laser Coheren Camaleon Vision II in mode locked (Coherent, Inc., Santa Clara, CA). Excitations were fixed at 730 or 980 nm for DAPI or PKH26, respectively. Fluorescent emission was collected using an NDD detector set in transmission mode using an SP 485 nm filter (DAPI staining) or with a BiG detector in reflection mode equipped with BP 420–475 nm for PKH26.

## Results

### Valves and cusps decellularization

**FIG. 1.**
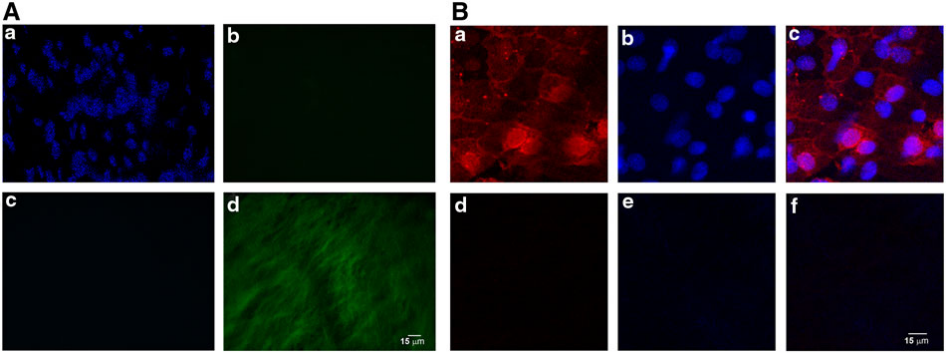
Native porcine endothelium observed by fluorescence microscopy and compared with the decellularized porcine scaffold. **(A)** Both native endothelium **(a)** and decellularized cusps **(c)** were stained by DAPI (blue). Autofluorescence of the matrix (green) was observed both on native endothelium **(b)** and on decellularized cusps **(d)**. **(B)** Native endothelium was stained by cell membrane labeling PKH26 **(**red, **a)** and DAPI **(b**, blue**)**. A PKH26 and DAPI merge image is also shown **(c)**. Decellularized cusps were stained by cell membrane labeling PKH26 **(**red, **d)** and DAPI **(e**, blue**)**. A PKH26 and DAPI merge image is also shown **(f)**. Images are representative of three separate experiments. Scale bar: 15 μm. DAPI, 4′,6-diamidino-2-phenylindole.

To decellularize heart cusps, two different detergent solutions, already reported in the literature, were compared: one composed of 1% SDS+0.05% NaN_3_,^4^ and another containing 1% Triton X-100.^17,27^ To prove the efficacy of the decellularization process, detergent-treated valves were observed by optical microscopy. Figure 1A-c evidences the complete suppression of the porcine endothelium by fluorescence microscopy since nuclei, pointed out by DAPI staining (blue) on the native cusps (Fig. 1A-a), disappeared when decellularized cusps were observed (Fig. 1A-c). In the green channel, the absence of cell nuclei (Fig. 1A-d) underlined the autofluorescence of the decellularized matrix; such a green autofluorescence disappeared when porcine valve are covered by the native endothelium (Fig. 1A-b). Data were confirmed by multiphoton microscopy analyses, showing the absence of the cellular component on the scaffold, after staining the decellularized valves with PKH26 (red) and DAPI (blue) (Fig. 1B-d–f); the native porcine endothelium was used as positive control for nuclei and membrane staining (Fig. 1B-a–c). Of note, the absence of PKH26-positive elements on decellularized cusps revealed that nor cells neither cellular fragments (which may induce immune reactions) were evidenced.

**FIG. 2.**
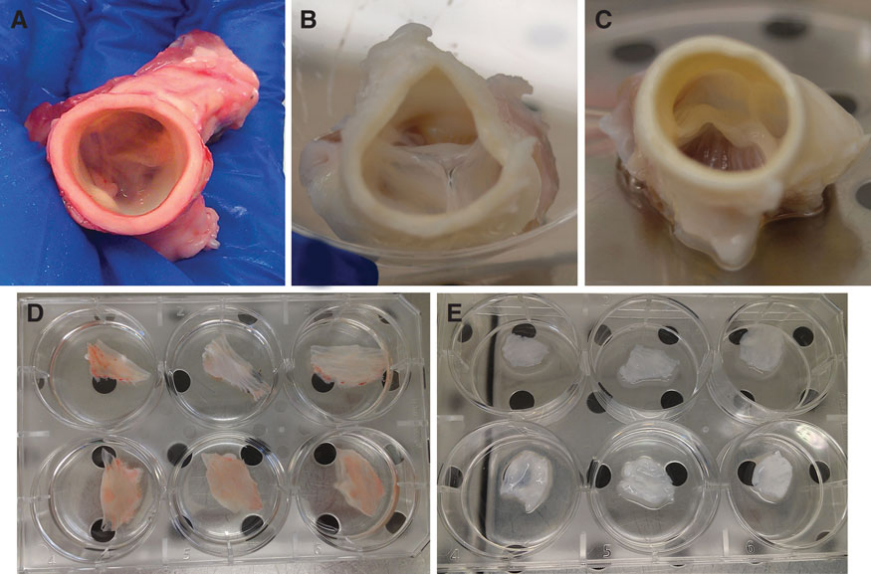
Entire porcine aortic valves decellularized with two different detergent solutions. Porcine aortic valve before **(A)** and after decellularization by 1% SDS+0.05% NaN_3_ **(B)** or by 1% Triton X-100 **(C)**. Dissected porcine aortic valve cusps before **(D)** and after decellularization by 1% SDS+0.05% NaN_3_ **(E)**. Images are representative of three separate experiments. SDS, sodium dodecyl sulfate.

Both aforementioned detergents gave similar results in terms of cell removal when compared to the morphology of the freshly harvested valve and cusps (Fig. 2A, D, respectively). Anyway, the decellularizing solution containing 1% SDS and 0.05% NaN_3_ determined a better maintenance of the valve structure (Fig. 2B) with respect to the corresponding Triton-treated specimens (Fig. 2B, C). For this reason, all the following experiments were performed by using only this solution. In any case, cusps were obtained and cultured (Fig. 2E).

Both aortic and pulmonary valves underwent the decellularization process; results demonstrated that the aortic scaffold better resist to the detergent treatment with respect to the pulmonary one (data not shown). Therefore, we decided to use only aortic roots for the following experiments.

### HUVECs and WJ-MSCs phenotype

**FIG. 3.**
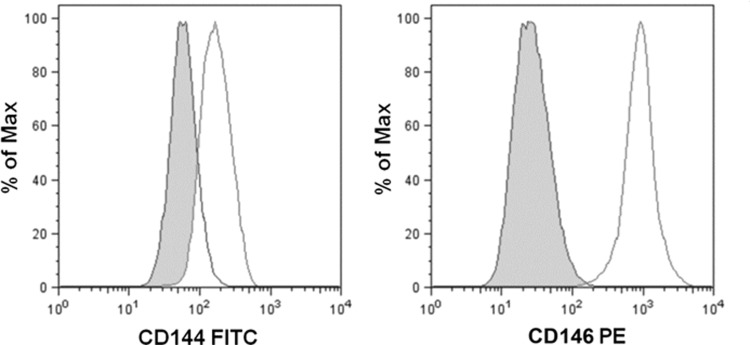
Flow cytometry immunophenotype analysis of HUVECs. Anti-CD144 FITC- or anti-CD146 PE-stained cells. The histogram shows the antigen expression (white curve) compared to the unstained control (gray curve). The image is representative of three separate experiments. FITC, fluorescein isothiocyanate; HUVEC, human umbilical vein endothelial cell; PE, phycoerythrin.

To ascertain the nature of both WJ-MSCs and HUVECs, isolated in our laboratories, flow cytometry phenotype analyses were carried out before their use in cusps recellularization experiments. As shown in Table 1, WJ-MSCs stained positive for surface markers typically expressed by MSCs (CD13, CD73, CD146, CD90, CD166, CD29, CD105, CD44, and HLA-ABC), while they did not express neither hematopoietic (CD14, CD34, CD133, CD45, HLA-DR) nor endothelial (CD31, CD144) or epithelial (ESA) markers. In addition, data analyses highlighted that HUVECs expressed both CD144 and CD146 endothelial markers on their surfaces (Fig. 3).

**Table 1.** **Flow Cytometry Analysis of Wharton's Jelly Mesenchymal Stem Cell Phenotype**

Antigens	Phenotype
CD13 FITC	+
CD14 FITC	−
CD29 PE	+
CD31 FITC	−
CD34 PE	−
CD44 FITC	+
CD45 FITC	−
CD73 PE	+
CD90 FITC	+
CD105 FITC	+
CD133 PE	−
CD144 FITC	−
CD146 PE	+
CD166 FITC	+
ESA PECy5.5	−
HLA-ABC Alexa488	+
HLA-DR PE	−

+, Positive marker expression; −, negative marker expression. Data are representative of three separate experiments.

FITC, fluorescein isothiocyanate; PE, phycoerythrin.

### WJ-MSCs endothelial differentiation

**FIG. 4.**
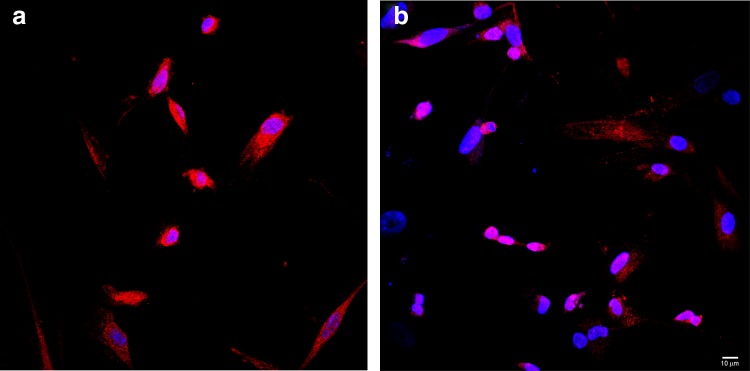
Differentiation of WJ-MSCs along the endothelial lineage. **(a)** WJ-MSCs were differentiated along the endothelial lineage for 15 days and were stained by DRAQ5 (blue), anti-CD31 and anti-mouse Alexa Fluor 546 (red). **(b)** WJ-MSCs were differentiated along the endothelial lineage for 15 days and were stained by DRAQ5 (blue), anti-CD144 and anti-mouse Alexa Fluor 546 (red). Images are representative of three separate experiments. Scale bar: 10 μm. WJ-MSC, Wharton's jelly mesenchymal stem cells.

After 15 days of culture under endothelial conditions, WJ-MSCs were stained for typical endothelial markers and observed by confocal microscopy. As shown, endothelial-differentiated WJ-MSC expressed both CD31 and CD144 (Fig. 4a, b).

### Cusps recellularization

**FIG. 5.**
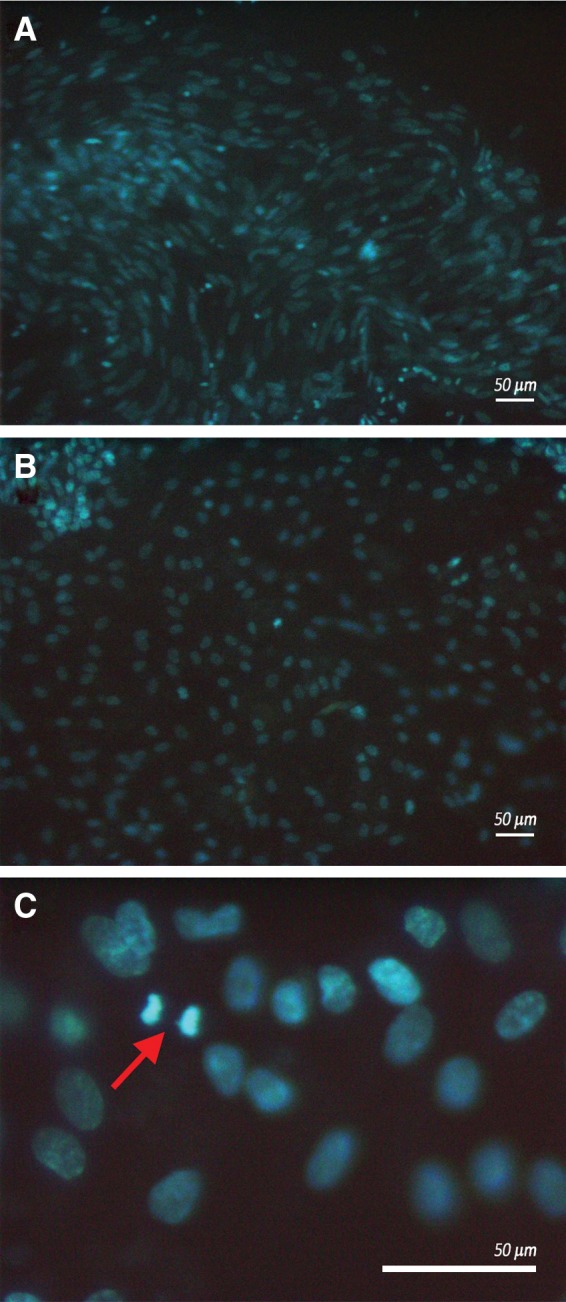
Porcine matrix was stained by DAPI (blue) and observed by fluorescence microscopy. Images refer to WJ-MSCs **(A)** and HUVECs **(B)**; WJ-MSCs retain their proliferation ability when adhering on the cusp matrix, as shown by the red arrow highlighting cells in metaphase **(C)**. Images are representative of three separate experiments. Scale bar: 50 μm.

**FIG. 6.**
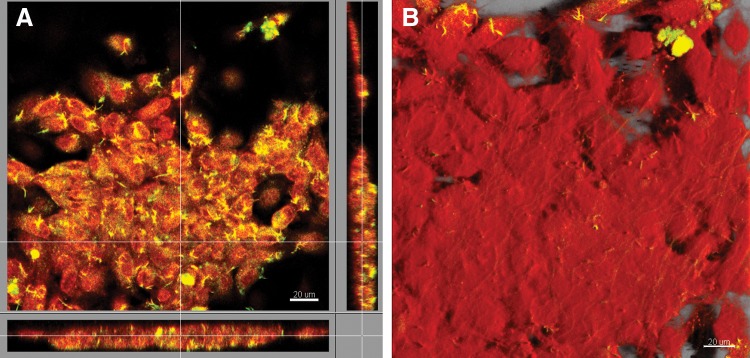
Confocal microscopy analysis of HUVECs. **(A)** HUVECs were stained by DRAQ5 (red) and CD146 PECy7 (yellow). Orthogonal projections show the organization of HUVECs in a cellular monolayer. **(B)** Three-dimensional isometric projection reconstruction; red color represent cell surfaces. Images are representative of three separate experiments. Scale bar: 20 μm.

Recellularization was performed culturing single decellularized cusps as substratum for differentiated-WJ-MSCs and HUVECs growth. After 15 days of culture, cusps recellularization was analyzed by fluorescence microscopy; data showed that both WJ-MSCs and HUVECs recolonized the cusps as demonstrated by the presence of nucleated adherent cells on the matrix (Fig. 5). In particular, WJ-MSCs and HUVECs, recellularizing the matrix, displayed characteristic patterns: a whirling cell growth for WJ-MSCs (Fig. 5A) and a uniform distribution for HUVECs (Fig. 5B) were observed. In both cases, cells retained their proliferative capacities; as shown in Figure 5C, the typical metaphase pattern (red arrow) suggested that cells went on dividing once kept in touch with the porcine matrix. Furthermore, the confocal microscopy analysis highlighted that both cell types, when growing on the porcine scaffold, were able to reconstitute a continuous cellular monolayer about 20 μm thick (Fig. 6A), establishing a close contact with the matrix fibers, as if they intercalate among them, as shown by the 3D reconstruction with the isometric projection (Fig. 6B). In any case, both HUVECs and differentiated-WJ-MSCs retained endothelial features when grown on the porcine scaffolds (data not shown).

## Discussion

The HVTE constitutes a promising approach to overcome the limitations related to the current surgical treatment of heart valve diseases, nowadays based on the implantation of biosynthetic or mechanical prosthesis.^6^ In this context, the reconstitution of functional, durable, and biological heart valve structures requires a deep evaluation of their basic elements (both matrix and cells).

In the present study, a natural scaffold (successfully obtained by decellularizing porcine heart valves) was used, demonstrating that it was able to positively interact with the cell compartment. Natural scaffolds provide bioactive components, ligands, and molecules (collagen, elastin, and proteoglycans),^27^ which are probably responsible for their ability, demonstrated here to induce cells to both adhere to the substratum and maintain their proliferative capacities. Ideally, the biological scaffold does not induce toxic degeneration and immunogenic or thrombogenic events, typical reactions often determined by synthetic polymer materials.^4,18,19^ For these reasons, it is likely that porcine scaffolds may contribute to the reconstitution of a long-term durable substitute.

The cell component represents another relevant element in the field of HVTE since it has been reported that the *in vivo* reimplantation of decellularized biological scaffolds induced their fast mechanical deterioration and degeneration.^28,29^ In the same way, cell sources need to be carefully chosen to guarantee both a long-term durability of the substitute and a low incidence of adverse reactions; therefore, WJ-MSCs seem to be ideal candidates in such a context. Our results strongly indicated that WJ-MSCs, differentiated along the endothelial lineage, as well as HUVECs (used as positive control), were able to adhere and proliferate when induced to interact with the porcine matrix, reconstituting a neoendothelium layer, characterized by typical endothelial features. The evidenced WJ-MSCs proliferative capacities, when in contact with a natural substratum, let us to hypothesize that they could reconstitute a pluristratified functional endothelium. Of note, differentiated WJ-MSCs demonstrated the ability to express typical endothelial adhesion molecules that normally do not characterize the mesenchymal phenotype; this suggests the tendency of MSCs to differentiate along the endothelial lineage. Anyway, further experiments will be needed to ascertain the maintenance of WJ-MSCs adherence on the scaffold in dynamic conditions. This evaluation may allow to translate the use of these valves in the clinical practice. In this context, it must be highlighted that WJ-MSCs are characterized by broad immunosuppressive properties. Indeed, it has been established that WJ-MSCs are able to inhibit lymphocyte proliferation and their soluble factor secretions. In particular, it has been shown that leukocyte IFN-γ production is reduced by WJ-MSCs^14^; TGF-β1, released by WJ-MSCs, inhibits T-lymphocyte activation and proliferation.^30^ Furthermore, WJ-MSCs downregulate many surface markers of monocyte-derived dendritic cells involved in inflammatory responses (i.e., CD1a, CD14, and the T costimulation complex CD86/CD80).^31,32^ For these reasons, the use of WJ-MSCs may also determine a low risk of a graft versus host disease development. Moreover, in the perspective of engineered and neoendothelialized cardiac valve regeneration, it must be underlined that it is possible to cryopreserve autologous WJ-MSCs at childbirth, therefore making them available in any moment of the life of the patient.

Altogether, these evidences introduce a novel idea: the “custom-made heart valve prosthesis,” which could real fit on the patient who needs valve substitution. If from one side, this concept could mean a ready availability of the prosthesis, on the other side, it could reduce the incidence of reinterventions, leading to a better quality of life for patients.

## Abbreviations Used

APC: allophycocyanin
BP: band pass
DAPI: 4′,6-diamidino-2-phenylindole
DPBS: Dulbecco's phosphate-buffered saline
DMEM: Dulbecco's modified Eagle's medium
ECGF: endothelial cell growth factor
FBS: fetal bovine serum
FITC: fluorescein isothiocyanate
HTF: HydroTech Filter
HUVECs: human umbilical vein endothelial cells
HVTE: heart valve tissue engineering
IFN-γ: interferon-gamma
MFI: mean fluorescence intensity
MSCs: mesenchymal stem cells
PE: phycoerythrin
PE Cy5.5: phycoerythrin-cyanine 5.5
P/S: penicillin/streptomycin
SDS: sodium dodecyl sulfate
TGF-β1: transforming growth factor-beta 1
WJ-MSCs: Wharton's jelly mesenchymal stem cells

## References

[1] Iung B, Baron G, Butchart EG, et al. A prospective survey of patients with valvular heart disease in Europe: the Euro Heart Survey on Valvular Heart Disease. Eur Heart J. 2003;24:1231–1243 12831818 10.1016/s0195-668x(03)00201-x

[2] Bonow RO, Carabello BA, Chatterjee K, et al. ACC/AHA 2006 guidelines for the management of patients with valvular heart disease: a report of the American College of Cardiology/American Heart Association Task Force on Practice Guidelines, 1st edn. J Am Coll Cardiol. 2006 10.1016/j.jacc.2014.02.536 24603191

[3] Bonow RO, Carabello BA, Chatterjee K, et al. 2008 Focused update incorporated into the ACC/AHA 2006 guidelines for the management of patients with valvular heart disease: a report of the American College of Cardiology/American Heart Association Task Force on Practice Guidelines, 1st edn. J Am Coll Cardiol. 2008;52:e1–e142 18848134 10.1016/j.jacc.2008.05.007

[4] Weymann A, Schmack B, Okada T, et al. Reendothelialization of human heart valve neoscaffolds using umbilical cord-derived endothelial cells. Circ J. 2013;77:207–216 23001070 10.1253/circj.cj-12-0540

[5] Kobayashi J Stentless aortic valve replacement: an update. Vasc Health Risk Manag. 2011;7:345–351 21731886 10.2147/VHRM.S11253 PMC3119592

[6] Weber B, Emmert MY, Hoerstrup SP Stem cells for heart valve regeneration. Swiss Med Wkly. 2012;142 10.4414/smw.2012.13622 22802212

[7] Langer R, Vacanti JP Tissue engineering. Science. 1993;260:920–926 8493529 10.1126/science.8493529

[8] Schmidt D, Hoerstrup SP Tissue engineered heart valves based on human cells. Swiss Med Wkly. 2006;136:618–623 17086507 10.4414/smw.2006.11400

[9] Yacoub MH, Takkenberg JJM Will heart valve tissue engineering change the world? Nat Clin Pract Cardiovasc Med. 2005;2:60–61 16265355 10.1038/ncpcardio0112

[10] Gerdisch MW, Shea RJ, Barron MD Clinical experience with CorMatrix extracellular matrix in the surgical treatment of mitral valve disease. J Thorac Cardiovasc Surg. 2014;148:1370–1378 24332188 10.1016/j.jtcvs.2013.10.055

[11] Ozeren M, Han U, Mavioglu I, et al. Consequences of PTFE membrane used for prevention of re-entry injuries in rheumatic valve disease. Cardiovasc Surg. 2002;10:489–493 12379408 10.1177/096721090201000508

[12] Umashankar PR, Mohanan PV, Kumari TV Glutaraldehyde treatment elicits toxic response compared to decellularization in bovine pericardium. Toxicol Int. 2012;19:51–58 22736904 10.4103/0971-6580.94513 PMC3339246

[13] Vono R, Spinetti G, Gubernator M, et al. What's new in regenerative medicine: split up of the mesenchymal stem cell family promises new hope for cardiovascular repair. J Cardiovasc Transl Res. 2012;5:689–699 22886691 10.1007/s12265-012-9395-2

[14] Zhou C, Yang B, Tian Y, et al. Immunomodulatory effect of human umbilical cord Wharton's jelly-derived mesenchymal stem cells on lymphocytes. Cell Immunol. 2011;272, 33–38 22004796 10.1016/j.cellimm.2011.09.010 PMC3235326

[15] Angelucci S, Marchisio M, Di Giuseppe F, et al. Proteome analysis of human Wharton's jelly cells during in vitro expansion. Proteome Sci. 2010;8:18 20346146 10.1186/1477-5956-8-18 PMC2867805

[16] Doan CC, Le TL, Hoang NS, et al. Differentiation of umbilical cord lining membrane-derived mesenchymal stem cells into endothelial-like cells. Iran Biomed J. 2014;18:67–75 24518546 10.6091/ibj.1261.2013 PMC3933914

[17] Di Tomo P, Canali R, Ciavardelli D, et al. β-Carotene and lycopene affect endothelial response to TNF-α reducing nitro-oxidative stress and interaction with monocytes. Mol Nutr Food Res. 2012;56:217–227 22162208 10.1002/mnfr.201100500

[18] Gorfien S, Spector A, DeLuca D, et al. Growth and physiological functions of vascular endothelial cells in a new serum-free medium (SFM). Exp Cell Res. 1993;206:291–301 8500549 10.1006/excr.1993.1149

[19] Lachmann R, Lanuti P, Miscia S OMIP-011: characterization of circulating endothelial cells (CECs) in peripheral blood. Cyometry A. 2012;81:549–551 10.1002/cyto.a.22071 22648996

[20] Lanuti P, Fuhrmann S, Lachmann R, et al. Simultaneous characterization of phospho-proteins and cell cycle in activated T cell subsets. Int J Immunopathol Pharmacol. 2009;22:689–698 19822085 10.1177/039463200902200314

[21] Lanuti P, Ciccocioppo F, Bonanni L, et al. Amyloid-specific T-cells differentiate Alzheimer's disease from Lewy body dementia. Neurobiol Aging. 2012;33:2599–2611 22330173 10.1016/j.neurobiolaging.2012.01.004

[22] Guarnieri S, Morabito C, Paolini C, et al. Growth associated protein 43 is expressed in skeletal muscle fibers and is localized in proximity of mitochondria and calcium release units. PLoS One. 2013;8:e53267 23308181 10.1371/journal.pone.0053267 PMC3538766

[23] D'Alimonte I, Lannutti A, Pipino C, et al. Wnt signaling behaves as a “master regulator” in the osteogenic and adipogenic commitment of human amniotic fluid mesenchymal stem cells. Stem Cell Rev. 2013;9:642–654 23605563 10.1007/s12015-013-9436-5 PMC3785124

[24] Sulpizio M, Falone S, Amicarelli F, et al. Molecular basis underlying the biological effects elicited by extremely low-frequency magnetic field (ELF-MF) on neuroblastoma cells. J Cell Biochem. 2011;112:3797–3806 21826706 10.1002/jcb.23310

[25] Dohmen PM, Konertz W Tissue-engineered heart valve scaffolds. Ann Thorac Cardiovasc Surg. 2009;15:362–367 20081743

[26] Grauss RW, Hazekamp MG, Oppenhuizen F, et al. Histological evaluation of decellularised porcine aortic valves: matrix changes due to different decellularisation methods. Eur J Cardiothorac Surg. 2005;27:566–571 15784352 10.1016/j.ejcts.2004.12.052

[27] Mayer JE Uses of homograft conduits for right ventricle to pulmonary artery connections in the neonatal period. Semin Thorac Cardiovasc Surg. 1995;7:130–132 7548318

[28] Schoen FJ, Levy RJ Founder's Award, 25th Annual Meeting of the Society for Biomaterials, perspectives. Providence, RI, April 28–May 2, 1999. Tissue heart valves: current challenges and future research perspectives. J Biomed Mater Res. 1999;47:439–465 10497280 10.1002/(sici)1097-4636(19991215)47:4<439::aid-jbm1>3.0.co;2-o

[29] Juthier F, Vincentelli A, Gaudric J, et al. Decellularized heart valve as a scaffold for in vivo recellularization: deleterious effects of granulocyte colony-stimulating factor. J Thorac Cardiovasc Surg. 2006;131:843–852 16580443 10.1016/j.jtcvs.2005.11.037

[30] Simon P, Kasimir MT, Seebacher G, et al. Early failure of the tissue engineered porcine heart valve SYNERGRAFT in pediatric patients. Eur J Cardiothorac Surg. 2003;23:1002–1006 12829079 10.1016/s1010-7940(03)00094-0

[31] Bommireddy R, Saxena V, Ormsby I, et al. TGF-beta 1 regulates lymphocyte homeostasis by preventing activation and subsequent apoptosis of peripheral lymphocytes. J Immunol. 2003;170:4612–4622 12707339 10.4049/jimmunol.170.9.4612

[32] Saeidi M, Masoud A, Shakiba Y, et al. Immunomodulatory effects of human umbilical cord Wharton's jelly-derived mesenchymal stem cells on differentiation, maturation and endocytosis of monocyte-derived dendritic cells. Iran J Allergy Asthma Immunol. 2013;12:37–49 23454777

